# Redefining the Human Oral Mycobiome with Improved Practices in Amplicon-based Taxonomy: Discovery of *Malassezia* as a Prominent Commensal

**DOI:** 10.1371/journal.pone.0090899

**Published:** 2014-03-10

**Authors:** Amanda K. Dupuy, Marika S. David, Lu Li, Thomas N. Heider, Jason D. Peterson, Elizabeth A. Montano, Anna Dongari-Bagtzoglou, Patricia I. Diaz, Linda D. Strausbaugh

**Affiliations:** 1 Center for Applied Genetics and Technology and Department of Molecular and Cell Biology, The University of Connecticut, Storrs, Connecticut, United States of America; 2 Division of Periodontology Department of Oral Health and Diagnostic Sciences, School of Dental Medicine, University of Connecticut Health Center, Farmington, Connecticut, United States of America; University of Wisconsin Medical School, United States of America

## Abstract

Fungi are a large, complex group, increasingly recognized as emerging threats. Their roles as modifiers of health mandate accurate portrayals of fungal communities in humans. As an entry point into the airways and gastrointestinal tract, fungi in the mouth are relevant to several biocompartments. We have revised current practices in sequence-based taxonomy assignments and employed the improvements to address the question of the fungal genera present in the healthy human mouth. The human oral mycobiome was surveyed using massively parallel, high throughput sequencing of internal transcribed spacer 1 (ITS1) amplicons from saliva following robust extraction methods. Taxonomy was assigned by comparison to a curated reference dataset, followed by filtering with an empirically determined BLAST E-value match statistic (10^−42^). Nomenclature corrections further refined results by conjoining redundant names for a single fungal genus. Following these curation steps, about two-thirds of the initially identified genera were eliminated. In comparison with the one similar metagenomic study and several earlier culture-based ones, our findings change the current conception of the oral mycobiome, especially with the discovery of the high prevalence and abundance of the genus *Malassezia*. Previously identified as an important pathogen of the skin, and recently reported as the predominant fungal genus at the nostril and backs of the head and ear, this is the first account of *Malassezia* in the human mouth. Findings from this study were in good agreement with others on the existence of many consensus members of the core mycobiome, and on unique patterns for individual subjects. This research offered a cautionary note about unconditional acceptance of lengthy lists of community members produced by automated assignments, provided a roadmap for enhancing the likely biological relevance of sequence-based fungal surveys, and built the foundation for understanding the role of fungi in health and disease of the oral cavity.

## Introduction

The fungi are among the most environmentally abundant and diverse eukaryotes, with estimates ranging from the more conservative approximation of 1.5 million species [1], [2] to as many as 5.1 million based on high-throughput sequencing methods [3]. Many of these organisms are not culturable outside of their specialized ecological niches, and only about 1–5% of the estimated species have been described. Fungi have been recognized as an emerging threat to animal and plant biodiversity [4]. More than 600 fungal species are reported to infect humans [5] and are associated with a wide range of diseases from skin conditions [6] to asthma [7]. The fungi are environmental opportunists that can occupy, and in some cases colonize, diverse biocompartments in individuals who come in contact with them in both outdoor environments where fungi are ubiquitous and occupy an amazing range of ecological niches [3], and indoor environments where fungi are found on food, in the air and on many surfaces (see, for examples [8], [9]). The emergence of fungi as a prevalent class of human pathogens has been a relatively recent occurrence, mapping temporally to the past several decades and thought to be facilitated by an increase in the number of immunocompromised persons, due to both widespread viral epidemics and medical interventions (discussed by Casadevall in [10]). Understanding their direct and indirect effects on human health requires a full characterization of fungal commensals and pathogens in both healthy and diseased populations.

Despite the widespread association of fungi with all plants and animals, and the importance of fungal communities to agriculture, biotechnology, ecosystem stability, and human health, they have remained relatively poorly characterized, due in large part to their recalcitrance to traditional methods of culture and identification. Recently, massively parallel, high-throughput sequencing has provided unprecedented genome-based views of the composition and diversity of fungal communities in human biocompartments [11]–[14], and ones that are not limited by requirements for culturing. Fungal sequencing efforts have been augmented by the availability of a pipeline for the analysis of 18S ITS sequences (CloVR-ITS) that introduced parallel capabilities to those existing for bacterial 16S amplicons [15]. Although metagenomic approaches generate comprehensive datasets, there are three fundamental challenges to extracting meaningful community profiles for fungi. First is the universal issue of the recognition of process-induced sequencing errors [16]. Second are the considerations of legitimacy and accuracy of taxonomic assignments that will define the community members and structure [17]. Third are the fungal-specific challenges to binary naming and phylogenetic classifications [18]. We focused on the latter two challenges as ones that affected both qualitative and quantitative conclusions about the composition of a given fungal community. Our aims were to empirically develop dataset curation guidelines with an awareness of the special mycological issues involved and then apply them to describe the human oral mycobiome. In doing so, we have addressed the three challenges posed earlier by answering the questions: Which sequences in our dataset likely represent artifacts rather than biological diversity? What computational measures of the strength of a match between experimental and reference database sequences reflects a probable authentic taxonomic assignment to fungi? What is the most appropriate nomenclature and best way to unify redundant taxonomic assignments for a given sequence? We describe herein the parameters we developed for assessment of fungal amplicon sequence assignments, and for deconvoluting nomenclature to arrive at a more meaningful genus level survey of fungal communities.

The long term goal of this research is to use genomic strategies to understand how changes in the microbiome, both bacterial and fungal, play roles in the development of oral diseases [19]–[21]. As a first step toward establishing the fungal baseline for this goal, we developed a robust workflow for DNA extraction, 18S ITS1 amplification, pyrosequencing, and curation, and applied it to analyze the fungal community composition in saliva samples from six healthy individuals. Several findings from our work expand the understanding of the human “core mycobiome.” First, we confirmed the following consensus genus-level members with the only other similar study on the oral mycobiome [11]: *Alternaria/Lewia*, *Aspergilllus/Emericella/Eurotium*, *Candida/Pichia*, *Cladosporium/Davidiella*, *Cryptococcus/Filobasidiella*, *Fusarium/Gibberella*, *Aureobasidium*, *Saccharomyces*, *Epicoccum* and *Phoma*. Second, and most strikingly, we discovered *Malassezia* species, previously only noticed as commensals and pathogens of the skin, as predominant commensals in saliva. Moreover, our findings provide guidelines of how to improve curation of the often lengthy lists of fungal community members produced by automated sequence assignments, thereby better harnessing their biological relevance.

## Results and Discussion

### Defining Parameters for Curation of Sequence Datasets

We used a pilot study of saliva samples (representing three subjects) with positive (*Candida albicans* gDNA) and negative (no DNA) controls to develop adequate DNA extraction methods and quality control procedures (Table 1). For sequence analysis, in addition to sequence processing (QIIME) and removal of human and bacterial sequences (DeconSeq), we added a custom program to remove primer artifacts. Sequences were submitted to the Fungal Metagenomics Project (FMP [22]) and taxonomic assignments used in analysis.

**Table 1:** Quality Controls.

Sample^a^	Total sequence count	Total minus primer artifacts (% previous column)	Sequences remaining after QIIME restrictions (% previous column)^b^	Sequences with no hits	# Genera assigned
**A. Pilot Studies**					
Saliva from 3 subjects	23,779	17,282 (72.7%)	15,627 (90.4)	735	222
**B. Negative Controls**					
No template added	2,245	22 (0.98%)	19 (86%)	0	8^c^
**C. Positive Control**					
*C. albicans* genomic DNA	63,069	63,043 (99.9)	60,282 (95.62)	4	6^d^

a One region of an 8 gasket PTP was used for a positive control (C). Negatives, positives, and pilot samples (representing a subset of three subjects) were sequenced in one region on the same run, and pilot l (A) and negative control (B) sequences partitioned by MID.

b QIIME restrictions: Minimum length  =  100 (after trimming forward primer and MID); maximum “N” = 1, maximum homocopolymer  = 10; maximum forward primer mismatch  = 2; maximum barcode mismatch  = 2.

c Genus assignments (sequence counts): Unclassified fungi (6 sequences); Saccharomyces (3); Tumularia (3); Malassezia (2); Rhodotorula (2); Candida (1); Ceratobasidium (1); Galerina (1). One of the genera assignments (Ceratobasidium) was at a very weak E-value (3.9); all others were at very strong E-values (−85 to −177).

d Includes *Candida*. All non-*Candida* genera are constituted by singleton sequences; 4 (*Scutellospora,Tomentella, Saccharomyces, Cryptococcus*) have very weak E-values (0.11–4.8); 1 (*Fusarium*) has a strong E-value (−119).

Applying the workflow derived from the pilot studies, we next generated the full experimental dataset that represents six new subjects. The resulting FMP assignments (Table S1) were used to develop parameters for curation of sequence datasets and formed the basis for the majority of our findings. The processing of all samples, from extraction through sequencing, was conducted under strict aseptic conditions in laboratory spaces specifically designed for forensic genetic typing, and maintained at that level of cleanliness and sterility.

#### Reduction of Primer Artifacts

Despite considerable efforts to optimize PCR amplification procedures, we observed that the specific conditions (samples, primers) we used resulted in primer artifacts ranging from dimers to larger and more complex multimers. These artifacts wasted sequencing space, introduced unnecessary computational demands, and confounded results. In an analysis of the length distribution of the full sequence dataset (data not shown), sequence lengths <99 bp were non-informative with only poor sequence alignments, and accounted for the vast majority of primer artifacts. However, some reference sequences composed of larger primer artifacts have been annotated in NCBI datasets as fungal, typically “unknown” or “uncultured”. An example of this was accession #GQ866198 which resulted in identification of a 244 bp sequence as an uncultured fungal clone (E-value 10^−41^), even though both the database reference sequence and our experimental sample sequence were composed exclusively of primer sequences. To avoid primer artifact complications, we adopted both pre- and post-sequencing measures. The length distribution data guided optimal choice of size selection cassettes for pre-sequencing removal of small amplicons and supported the exclusion of post-filtering sequences of less than 99 bases. In addition, we wrote a program that recognized and specifically removed sequences of any length that were exclusively derived from concatenated primers (Table 1, column 3).

#### Curation of sequence datasets based on abundance

A key step was to determine the count abundance that would avoid mistaking sequencing errors for biological variation. Negative and positive controls were used to empirically develop parameters for the minimization of genera that were artifacts (Table 1B, C). For both negative and positive controls, a very low number of erroneous sequences and genera were obtained. Furthermore, for the 12 genera incorrectly identified as present, each was represented by 3 or fewer sequences, and 8 were singleton assignments. Of these 12 genera, 7 identifications (*Candida, Galerina, Malassezia, Rhodotorula, Saccharomyces*, *Tumularia*, and *Fusarium*) had very strong E-values and 5 (*Ceratobasidium, Scutellospora, Tomentella, Saccharomyces, and Cryptococcus*) had very weak ones. All genera except *Galerina* were found in the full 6-subject experimental dataset (Table S1). For curation, we adopted a conservative threshold of 4 sequence counts for exclusion of rare genera, in good agreement with the minimum number of 5 sequences adopted in a study of the fungal communities in the human airway [12], and even more stringent than the recommended conservative approach of removing all singletons to prevent taxon overestimation and contamination with artifacts [16]. Although our count threshold eliminated only a small fraction of the total sequences, the impact on the number of genera was dramatic. Count-based exclusion of assignments from the first pass experimental sequence set removed 34% of the genera assignments, more than half of these were represented by sequence singletons (Table S1). Whether unexpected sequences or genera in controls resulted from spill-over from adjacent experimental samples or process-induced errors in sequencing [16], the results raised a cautionary note about the meaning of unclassified fungi since 24% (6/25) of known errors were reported as such.

### Developing Supplemental Parameters to Improve Taxonomic Identification

Because BLAST-based programs without well-developed reference sequences may use short sequences to force a taxonomic identification, it was important to understand the difference between constructive assignments and inconsequential ones. Insignificant assignments could arise from artifact sequences due to errors in amplification and sequencing, as well as from poor matches of authentic sequences to inadequate reference sets. One advantage of the Fungal Metagenomics Project (FMP) was that its database is curated weekly to contain only fungal reference sequences, specifically excluding “uncultured” and “environmental” descriptors. To illustrate this point, the most abundant sequence in our full experimental dataset has been assigned to the genus *Malassezia* from the curated FMP for the past two years (Table S1); but was designated as “uncultured fungus clone” by NCBI until March 2013. As a consequence, a prominent community member was concealed by its inclusion in an indefinite category. The FMP is a large database, drawn from GenBank, AFTOL and TreeBASE, and as a result both E-values and bit scores are reliable statistics. Even with the use of curated reference sets, the problem of poor assignments still remained, so we analyzed the first pass taxon assignments (Table S1) to develop additional E-value parameters to improve the recovery of legitimate fungal assignments.

#### Spurious taxonomic assignments can confound results and interpretations

Table S1 depicts the taxonomic assignments for the full experimental sequence set and illustrated the kinds of spurious assignments that occurred. For example, the genus *Lysurus*, a saprobic fungus commonly known as the lantern stinkhorn, and not expected in human saliva, was identified (row 132) with a median alignment value of 3.00E-20 and a poor bit score of <100, an assignment driven by the 18S and 5.8S portions of the sequence (Table S2). NCBI BLAST analysis of the entire sequence yielded matches to numerous melon sequences at E-values of −40 to −145. *Lysurus* was clearly not an authentic oral fungal community member, but rather an example of a non-specific food-derived sequence mistakenly assigned fungal identity. Fungal identities were inappropriately ascribed to other common dietary plant-derived sequences (from Table S1, row #; from Table S2, unmasked E-value for fungal assignment, designated by an asterisk, followed by E-values for plant matches): *Hygrophorus* (#38, E-20*, E-42), *Moniliella* (#46, E-16*, E-64), *Boletus* (#169, E-21*, E-123 to -148), *Pseudozyma* (#227, E-17*, E-165), and *Podosphaera* (#393, E-17*, E-172). These observations indicated that matches with a random chance of >1E-21 were more likely to represent inauthentic fungal assignments. The fact that the E-values for the incorrectly assigned fungal identities were within the range often deemed acceptable provided another dimension to the need for caution in the acceptance of automated ITS-based taxonomic assignments [23]. Identification of plant species with fungal primers was not unique to our results, and was also reported in studies of a human stool sample [24]. As noted by others [16], the primer used in this study (ITS1F) provided strong recovery of fungal amplicons, but also amplified trace amounts of plants. While simply masking the conserved regions offered the advantage of minimizing the incorrect assignments of plant-derived sequences, it had the disadvantage of eliminating those fungi, either described or undiscovered, that have no reference sequence and therefore no valid taxonomic assignments.

#### BLAST parameters can aid in distinguishing biological relationships from chance occurrences

While recent oral mycobiome studies have used alignment identity thresholds (generally 97–98%) to assign species identifications to ITS1 sequences, the suitability of this practice has been questioned [23]. We found that this standard resulted in reductions in representation of taxa that were abundant, frequent and potentially biologically meaningful. As an example, of the 18,914 sequences assigned to *Emericella nidulans* (Table S3), an alignment threshold of 97% eliminated 13,601 (72%), leading to an underrepresentation of a known opportunistic pathogen. Thresholds of 90% are sometimes used for genus-level identifications, but these also have potential problems. We investigated E-value thresholds as a supplemental identification metric by mapping increasingly stringent thresholds (representing an arbitrary doubling, tripling and quadrupling of the exponent) onto a subset of sequence assignments for a single subject (#50, the most diverse individual sampled in our study, Table S3).

**Figure 1:**
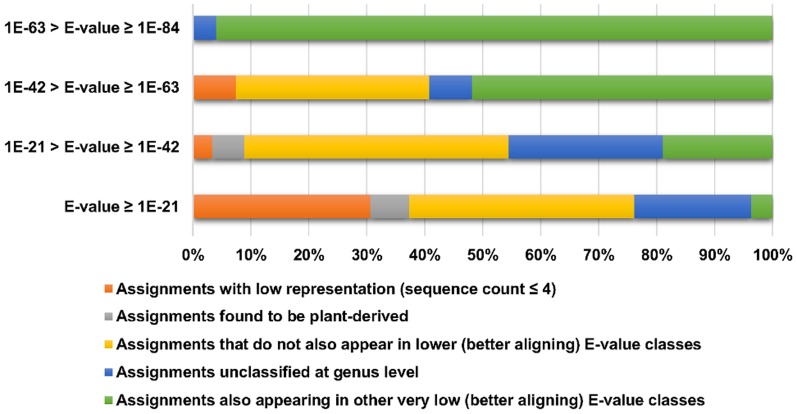
Increasingly Rigorous E-value Intervals Maximize Recovery of Authentic Fungal Assignments. Bars depict relative proportion of total sequence assignments in that interval that are accounted for by each color-coded category: low abundance representation (orange); plant-derived incorrect assignments (gray); occurrence only in that or a weaker E-value interval (yellow); assignments at class or order levels (blue), and genus assignments that are also included in other very low E-value assignments (green).

Figure 1 summarizes the effects of increasing E-value thresholds. The least restrictive E-21 removed 134 genera assignments, representing sequences that failed to meet the minimum count rule and plant derived sequences. Other assignments in this interval were to the genera *Neopaxillus*, *Mortierella*, and *Ramicandelaber* and were characterized by poor E-values (>−21) driven by 18S and/or 5.8S alignments; these three taxa appeared in only marginally better intervals at E-24. Only 4% of the sequences removed by this threshold represented genus level assignments also identified by much stronger matches in the same individual (e.g. *Saccharomyces, Pichia, Cordyceps, Cortinarius*). We concluded that there was no loss of fungal genera by imposing the E-21 threshold in curation of the taxon assignment dataset. At the most restrictive interval of E-64 to E-84, neither sequences below the abundance threshold (4 counts), nor sequences derived from plants were present. Assignments that did not reach genus level resolution were present, but minimal (4%). The vast majority of genera assignments (96%) were also included in even stronger E-values, lending support to the conclusion that taxonomic assignments in this interval represented authentic fungal components. As evidence of assurance in taxon identifications, 99% of the sequence assignments in this entire dataset (#50) were stronger than the E-63 restrictive threshold, and 97% met an E-95 threshold suggested previously as a basis for confidence in genera assignments [25].

Our goal was to choose a threshold from intermediate intervals that would achieve both confident identifications and “conservative flexibility” for natural variants. In the E-22 to E-42 interval, sequences below the count thresholds and ones based on plant identities remained; almost half (46%) of the sequence assignments were to three genera with no E-value stronger than E-42 in the entire sequence set. Included in this group were the plants *Osmorhiza* (NCBI BLAST match to carrot, Table S2) and *Tilia* (NCBI BLAST match to tomato, Table S2). The genus *Coniosporium* (a rock-inhabiting fungus) was assigned to sequences in this interval based on a poor alignment; however, based on strong alignment and E-value to an uncultured fungus, this most likely represented a legitimate fungal sequence that cannot be assigned with confidence to any genus based on current reference databases. About a quarter of the sequence assignments (27%) were to the less precise phylum level (Basidiomycetes, endophytic). The percentage of sequence assignments to genera also included in much stronger E-values (−52 through −135) increased to 19%, distributed over five genera (*Candida*, *Cortinarius*, *Cryptococcus*, *Mycosphaerella*, and *Pichia*). In the next most stringent interval of E-43 to E-63, plant-derived assignments disappeared and phylum/class assignments were reduced, all to the Dothiodiomycetes. More than half (52%) of the sequence assignments were to five genera that were also included in much stronger E-value groups, *Candida, Malassezia, Mrakia, Mycosphaerella*, and *Pichia*. Based on these findings, we adopted an E-value threshold of ≤−42 for inclusion in the curated assignment list. We also note that the alignments that met this threshold had bit scores that were ≥ the 200 bit score filter adopted in a study for ITS2 amplicons [12]. We confirmed the validity of our E-value threshold by evaluating its performance against sequence sets for all five remaining subjects. Whether the subject represented individual variation similarly to the highly diverse subject 50 or a less diverse community such as subject 51, the results were still fully consistent with the details provided in this section with respect to the kinds of inappropriate taxa that were eliminated. The finding that E-values significantly lower than those routinely deemed as acceptable could still represent spurious assignments to fungal genera is an important one that can result in misleading interpretations about fungal community members. An intriguing aspect of the E-value threshold is that this single filter effectively removes low abundance representation, plant-derived amplicons, unclassifiable sequences, and those identifications based on short conserved sequences or otherwise poor alignments.

#### Consequences of applying curation rules

**Figure 2:**
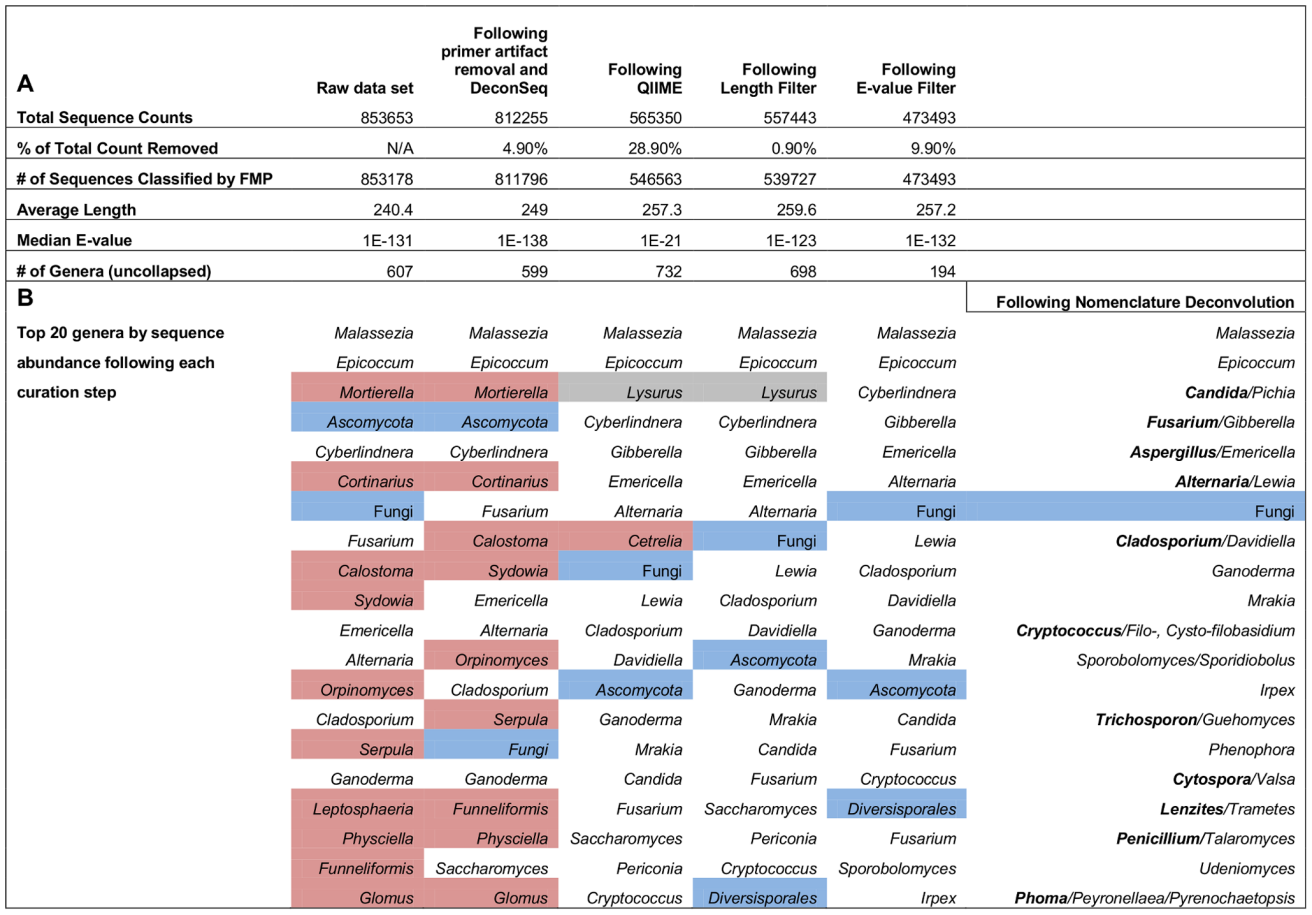
Stepwise quantitative and qualitative impact of application of curation rules. Panel A illustrates the effect of stepwise application of curation rules on the number and characteristics of sequences that are retained after each step (rows 1, 2, 4), and on the classifications by the Fungal Metagenomics Project (rows 3, 5, 6). Panel B depicts the changes to the top 20 taxa as a result of each step in the curation: gray cells depict plant-derived sequences incorrectly assigned fungal identity; blue cells depict classification at a higher taxonomic level than genus; red cells depict weak assignments driven by short conserved sequences.

Part A of Figure 2 illustrates the stepwise consequences of applying our curation rules to the full experimental sequence dataset. At the end of all curation steps, 55% of the original sequences remained, all of which were classified by the FMP. The QIIME step accounted for the removal of the majority of sequence classifications (18,757), followed by the E-value filter and primer artifact/DeconSeq screens. Since the QIIME trimming step removed about 40 bases of highly conserved universal primer sequence (as well as the MID tags), the alignment relied more on the informative ITS1 variable regions and resulted in both weaker median E-values and more genera assignments. Compared to the original assignments (Table S1), about 2/3 of the genera identified were eliminated following all curation steps. The E-value filter accounts for almost all of the effect on number of genera. The sequence curation eliminated several genera identifications that were perplexing as members of a human biocompartment based on their previously described ecological and geographical considerations.

Stepwise application of curation rules had a dramatic effect on the top 20 most abundant taxon assignments (Part B of Figure 2). While *Malassezia* and *Epicoccum* remained ensconced at the top throughout, the rest of the ranked list displayed movement. The top two fungal genera identified, *Malassezia* and *Epicoccum*, provided high benchmarks for authentic fungal identifications in the data set. The vast majority of alignments assigned to *Malessezia* had very strong E-values (median −163) and bit scores (206–632). Similarly, those assigned to *Epicoccum* also had very strong E-values (median −120) and bit scores (406–468). When the full set of curation rules were applied, low abundance representation (orange), plant-based incorrect fungal assignments (gray) and identifications based on poor alignments (yellow) disappeared, as did many of the assignments that could not be made to the genus level (blue). These results also provided an opportunity to illustrate the usefulness of the E-value screen. Sequences assigned to the genus *Serpula* appeared in the raw data set, but were reclassified as *Lysurus* following QIIME; however, these assignments failed the E-value threshold and were plant-derived, as discussed in a prior section.

While the elimination from further consideration of plant-based assignments with poor fungal E-values was both warranted and likely a permanent exclusion, there was a stipulation for other assignments. Lack of a robust fungal identification or failure to achieve a genus-level assignment could have reflected limited content and/or annotation in reference databases. Figure S1 summarizes the level of taxonomic assignment for those sequences that could not be assigned to the level of genus. While few were assigned to the family level, most were assigned with confidence to orders. Since the NCBI sequence databases are constantly expanding, and the FMP, like other reference databases, is regularly updated and refined, identification must remain an ongoing and evolving process. Most of the sequences with weak E-values (<E-21 but >E-42) contain conserved flanking 18S or 5.8S sequences, and those that cannot be assigned to the genus level at this time should be periodically reanalyzed for more robust taxonomic assignments as databases are perfected. One of the more significant findings of this analysis was the likelihood that studies using commonly accepted E-value thresholds identified fungal community members that may not be authentic.

### Nomenclature Deconvolution

Genera identified in the top 20 rankings also provided an opportunity to consider the challenges that nomenclature posed to the curation of taxon assignment datasets, subsumed under the “1N = 1F” (One Name One Fungus) initiative. We focused on genus level assignments because they represented very strong probabilities of non-random matches, and most of the taxonomic assignments in this level were derived from multiple reference sequences, often including type species. We collapsed genus assignments by considering alternate names, common knowledge of the teleomorph (sexual form) and anamorph (asexual form) pairs, previously published recommendations, and the more specific taxonomy assignment in our dataset. We also created our own biblioinformatic examination of “common usage” (Table S4) as suggested by Hawksworth [26]. Our usage table was based on assignments in our own dataset, and is by no means comprehensive. In the nomenclature deconvolution process, we referred by necessity to species names as well as genera in considering sexual/asexual pairs. Moreover, given the human and biomedical orientation of this project, we added NIH NCBI publications to our biblioinformatic metrics, as well as consideration of those genera known to be common oral inhabitants, in deciding which genus to list as the “priority” one in conjoined groupings. In order to avoid the loss of information inherent in dual nomenclature [27], we listed major constituents of the conjoined genera.

#### Collapsing genera into groupings conjoined by nomenclature

**Figure 3:**
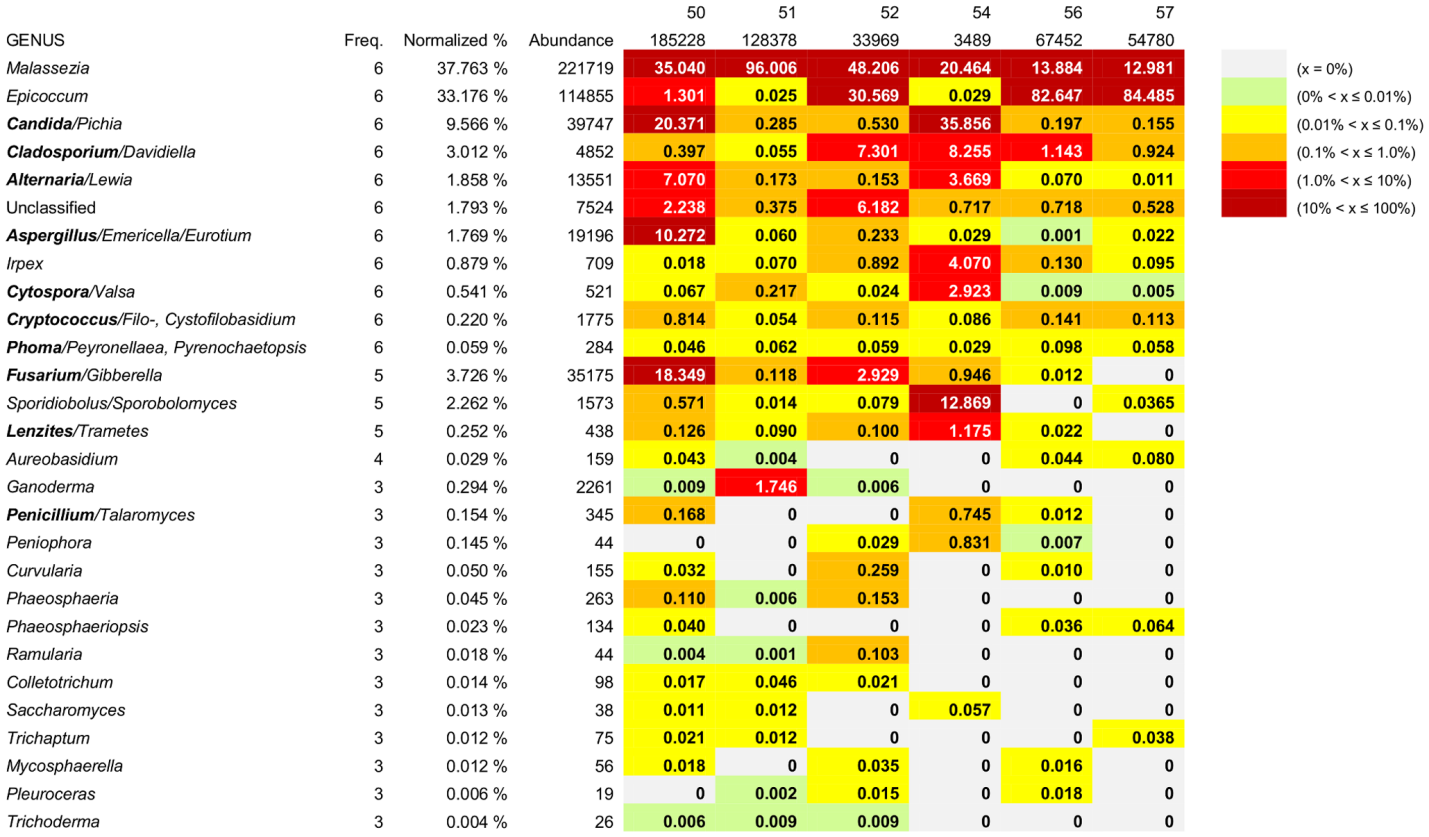
Frequency, abundance, and distribution of genera occurring in at least 50% of the six subjects. Genera ordered by frequency of occurrence, with normalized representation and sequence counts (columns 2, 3, 4). Heatmap depiction (columns 5–10) summarizes qualitative and quantitative distribution of genera in six individuals (50, 51, 52, 54, 56, 57) and depth of sequencing for each subject (row 2). Values within individual heatmap cells are the percentage representation within that subject.

Of the 17 genera listed in the fully curated top 20 (Figure 2, Panel B, Column 6), 12 were affected by nomenclature deconvolution. The genus *Cyberlindnera* was exclusively represented by its synonym, *Pichia jadinii*, so the former sequence counts were attributed to the genus *Pichia*. In turn, the genus *Pichia* was represented by three species: *jadinii, kudriavzevii*, and *membranifaciens*, all of which have the other names of *Candida utilis, Candida krusei*, and *Candida valida*, the respective anamorph forms. The *Pichia* sequence assignments were collapsed into *Candida*; the pair accounted for 0.2%–36% of sequences in individual subjects (Figure 3), a range in good agreement with the previously published study of Ghannoum and collaborators. While not every described species in the genus *Pichia* has a *Candida* counterpart, all *Pichia* identified in our sequence study did and were therefore appropriately combined. Across the six subjects in our study, sequences assigned to *Pichia* represented 99%, 43%, 81%, 7.7%, 6.7% and 0% of the combined *Candida* plus *Pichia* sequences (Figure 3). The teleomorphic genus *Gibberella* was often accompanied by its anamorphic genus *Fusarium* at identical E-values in the top 4–5 NCBI BLAST hits. In the vast majority of these cases, there were no species assigned to *Gibberella*, but assignments to *Fusarium culmorum* were common. In other *Gibberella* assignments, the species have *Fusarium* anamorph pairs. *Gibberella* sequence assignments were collapsed into the genus *Fusarium*. The genus *Emericella* was exclusively represented by the species *nidulans*, a synonym of *Aspergillus nidulans*, so the former sequence counts were attributed to *Aspergillus*. Assignments to genus *Eurotium* were also reassigned to *Aspergillus*, its priority genus [28]. Likewise, since the genus *Lewia* was exclusively represented by the species *infectoria*, the teleomorph form of *Alternaria infectoria*, we collapsed these sequences into *Alternaria*. The synonymous teleomorph genus *Davidiella* was collapsed into its anamorph genus *Cladosporium* [26].

Nomenclature considerations for less abundant taxa also affected the top categories (Figure 2, Panel B, Column 7). The teleomorph genera *Filobasidium (F. floriforme)* and *Cystofilobasidium (C. macerans)* were collapsed into the more commonly used nomenclature of its paired anamorph genus *Cryptococcus* [26]. *Trichosporon* was represented by the species *pullulans*, another name for *Guehomyces pullulans*; both sequence assignments were included under the genus *Trichosporon* (common usage). The anamorph species *Cytospora chrysosperma* (also called *Valsa sordida*) and *Cytospora translucens* were combined into the teleomorph genus *Valsa*. The genera *Lenzites, Penicillium*, and *Phoma* also rise in the listings by cumulative abundances. Many other assignments that were also affected by nomenclature deconvolution, but not in the top 20, are included in Table S4.

### Redefining the Human Basal Oral Mycobiome

In the pioneering, and to date only, metagenomic study of the human oral mycobiome published in 2010 by Ghannoum and colleagues [11], the threshold for considering a taxon (sequence) as a member of the basal mycobiome was occurrence in 20% of the individuals examined at an abundance of at least 1% of the sequences. We did not set any threshold for sequence abundance, in recognition that a community member widely present, but represented by a low percentage of the sequence counts, was still important to document. Figure 3 summarizes our findings for the genera present in at least 50% of our subjects (frequency≥3). The figure also includes the normalized representation, total sequence counts, and a heat map that shows the relative abundance of each genus in each individual. The heat map analysis fully confirmed the findings of others that subjects have individualized fungal profiles that differed from one another both qualitatively and quantitatively. Ghannoum and colleagues [11] identified thirteen taxa as core components of the human basal oral mycobiome; in our dataset, this number of genera was achieved by considering taxa present in more than half of our subjects (Figure 3), so we have used this subset for comparison.

#### Revisiting the Core Members

**Figure 4:**
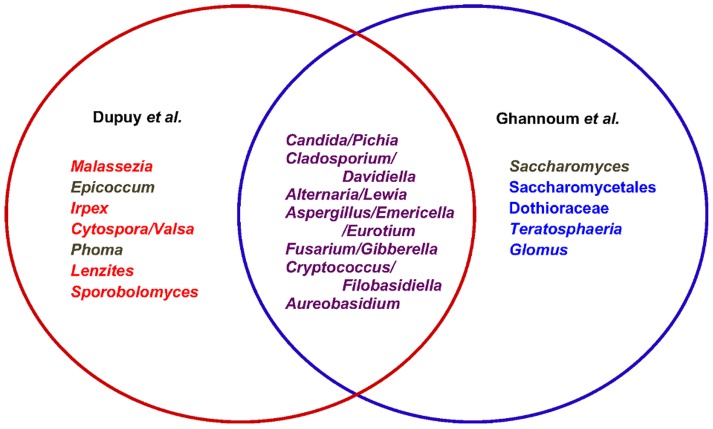
Venn diagram of the relationships between results from the two studies of the human oral mycobiome. Shared genera are indicated in the overlap (purple font) between the current study (Dupuy *et al*., red font) and the previously published study (Ghannoum *et al*., blue font). Genera in brown are shared between the two studies but failed to meet thresholds in one or the other.

The comparison between the results of Ghannoum and colleagues and our study is summarized in Figure 4. Ghannoum and colleagues reported thirteen components in the basal mycobiome: *Alternaria, Aspergilllus, Aureobasidium, Candida, Cladosporium, Cryptococcus*, Dothioraceae, *Eurotium, Fusarium, Glomus, Saccharomyces*, Saccharomycetales, and *Teratosphaeria*. Of the eleven that were identified at the genus level, our study also found eight of these in more than half of the subjects (genus followed by frequency and range): *Alternaria/Lewia* (100%, 0.01–7.07%), *Aspergilllus/Emericella/Eurotium* (100%, 0.001–10.27%), *Candida/Pichia* (100%, 0.12–35.86%), *Cladosporium/Davidiella* (100%, 0.06–8.26%), *Cryptococcus/Filobasidiella* (100%, 0.05–0.81%), *Fusarium/Gibberella* (83%, 0.01–18.35%), and *Aureobasidium* (67%, 0.004–0.08%). The genera *Saccharomyces* (50%), *Epicoccum* and *Phoma* were also shared, but were below thresholds in one study or the other. *Epicoccum* is found in indoor house dust samples [29], has been identified in air samples in buildings, including in the Northeastern U.S. where all of our subjects lived [9], and is a well-known air allergen. *Phoma* and *Epicoccum* were also identified as components of indoor fungal composition in temperate zones [8], and may represent environmental acquisitions specific to geography. While *Epicoccum* has not been associated with human infections, it has been identified as a source of allergens, and some species possess antifungal activity against pathogenic plant fungi. *Phoma* species were found to be causative of infection in a transplant recipient [30].

Four components of the core mycobiome proposed by Ghannoum and colleagues (*Glomus, Teratosphaeria*, Saccharomycetales and Dothioraceae) were absent from our high frequency listing. Several explanations could account for such discrepancies. First, the fungi were simply not present in the subjects sampled. Second, the identifications were spurious ones. Third, the taxonomic assignments were made to different levels in the two studies. In our case, sequences initially assigned to the genus *Glomus* were found in high abundance, but were eliminated following primer artifact and DeconSeq filters (Figure 2). Although *Teratosphaeria* was not detected in any of our subjects, we did have unclassified sequences in the order to which it belongs, Pleosporales (Figure S1). Two of the taxonomic assignments in the study by Ghannoum *et al.* were at the higher taxonomic ranks of order (Saccharomycetales) and family (Dothioraceae). Saccharomycetales is a large monophyletic order with about 1,000 known species across many genera [31], several of which were identified in our study. We note that one member genus in the family Dothioraceae, *Sydowia*, was a prominent taxon assignment in our study (Table S1 and Figure 2) before being eliminated by early step sequence curation filters (Figure 2).

Five genera (frequencies and ranges) were identified in high frequency in our study (Figure 3), but were not part of the basal oral mycobiome proposed by Ghannoum *et al*.: *Malassezia* (100%, 12.98–96.01%), *Irpex* (100%, 0.02–4.07%), *Cytospora/Valsa* (100%, 0.005–2.92%), *Lenzites/Trametes* (100%, 0.02–1.18%), and *Sporobolomyces/Sporidiobolus* (100%, 0.01–12.87%). *Malassezia* is discussed in the following section; all of the other four genera are common soil and/or plant pathogens that are widespread in common environmental sources in temperate zones. Members of three of these genera, *Irpex* [32], *Cytospora/Valsa* [33]and *Sporobolomyces/Sporidiobolus* [30] were previously identified as causative agents in infections in immune compromised persons. In this context, it seems prudent to consider these taxa worthy of attention in future studies.

#### The case for Malassezia as a new and predominant member of the basal oral mycobiome

The most unexpected finding from our study was the presence in all six subjects, at high abundances from 13% to 96%, of the genus *Malassezia* (Figures 2 and 3), one that was not identified by Ghannoum *et al*. [11]. On the experimental side, the results from our negative controls strongly support the conclusion that *Malassezia* sequences were not introduced during the processing of samples. There is additional support from the literature for the argument to include *Malassezia*, a recognized commensal and pathogen in humans and other mammals [34], as a member of the basal mycobiome. Well known to cause a variety of skin disorders [6], *Malassezia* was recently identified by metagenomic sequencing as associated with scalp disorders such as dandruff [13]. More directly relevant to the oral cavity, one of the main entryways for microbes into the airways, *Malassezia* was also discovered by metagenomic sequencing in the sputum of cystic fibrosis patients [12]. The mouth is the point of entry into the gastrointestinal tract, and *Malassezia* was identified by culture-independent, Sanger sequencing of cloned 18S amplicons from human stool [24]. Directly relevant to the mammalian oral cavity, *Malassezia* species were shown to occupy the mouth of dogs [35] and underwent zoonotic transfer by health care professionals from their dogs to neonates where they were responsible for serious infections [36]. Since the four more recent and culture-independent metagenomic studies that identified *Malassezia* in human biocompartments used subjects from worldwide geographies, different protocols for molecular biology, and different rules for sequence and taxon curation, the consensus on this genus was compelling evidence for its inclusion as a legitimate member of the basal oral mycobiome. It is noteworthy that each of the metagenomic studies reporting *Malassezia*, including ours, employed relatively harsh extraction protocols that were more likely to recover *Malassezia* DNA since species in this genus are known to have especially thick cell walls. The relatively harsh bead breaking step we employed did not appear to unfavorably impact recovery of other salivary genera given the agreement between our study and the prior report on the salivary mycobiome [11].

Additional characteristics of *Malassezia* species (reviewed in [34]) have probably contributed to the previous failures to recognize *Malassezia* as a prominent oral commensal. First, culture-based methods may not have captured *Malassezia* species since most have growth requirements for lipids and require specialized culture media [37]. Second, the taxonomy and nomenclature issues also complicated studies of *Malassezia*, which are dimorphic fungi (yeast and mycelial phases) that have been placed in multiple genera. Although much of the taxonomy within the genus has been sorted out, studies undertaken before the mid-1990s and those without knowledge of the recent resolutions of nomenclature may have missed this genus.

## Conclusions

A prime directive for understanding the role of fungal communities in human health and disease, as well as for making informed decisions about the nature and timing of medical intervention, is an accurate description of the fungi present. This depended on several factors. First, it is likely that the methodological protocol for initial breaking of cells and nucleic acid extraction had an important effect, leading to the identification of an otherwise intractable community member, *Malassezia*. Second, and in agreement with the conclusions of many others, the curation of sequence datasets prior to taxonomic analysis proved important. Our abundance threshold (4 counts for taxon inclusion) was very powerful in removing taxa of questionable validity, eliminating two-thirds of the genera, half of which were singletons. The alternative metric based on a relatively stringent E-value threshold that we developed for assessing legitimacy of the automated taxon assignments reduced spurious identifications. Experimental evaluation of sequence parameters also raised a cautionary note about the meaning of unclassified fungi, since both methodological and database errors were likely to be identified as such.

For more than two centuries, biologists have struggled with the challenges of the binary naming and phylogenetic classification of fungi. The challenges of multiple names for a single organism based on different stages or ecosystem origins has been exacerbated by genomic approaches and have been an ongoing topic of discussion in the mycology community [26], [38], [39]. We adopted a biblioinformatic process for managing nomenclature, recommended as an intermediate measure, pending a more full resolution in future years. Our decision to list components in nomenclature-based sets will permit future recommendations to be applied to the results. As we demonstrated, failure to appropriately collapse genera resulted in a much more complex list of community members and a severely misleading view of the relative abundance of some. A concrete example of the kinds of complications arising due to nomenclature is the recent conclusions reached in a comparison of fungal communities in healthy and dandruff-afflicted human scalps [13]. Park *et al.* [13] concluded that a genus-shift from *Cryptococcus* species to *Filobasidium* species characterized healthy versus dandruff-afflicted conditions, based on a dramatic increase in *Filobasidium floriforme*. The problem with this conclusion is that *Filobasidium floriforme* is a synonym for *Cryptococcus albidus*, and other *Filobasidium* species are likely sexual stages of *Cryptococcus* species. Accurate qualitative and quantitative information is especially imperative in biomedical applications where treatments may be involved. We found that some specific nomenclature research and hand curation were needed to create a more concise and meaningful survey of the various fungal communities associated with health and well-being.

This study confirmed nearly every community member described in the only similar study on the oral mycobiome (Ghannoum *et al*., 2010), despite different extraction protocols, analysis methods, and samples. Consensus members of the basal human salivary mycobiome were *Candida/Pichia, Cladosporium/Davidiella, Alternaria/Lewia, Aspergillus/Emericella/Eurotium, Fusarium/Gibberella, Cryptococcus/Filobasidiella*, and *Aureobasidium. Saccharomyces, Epicoccum* and *Phoma* were weaker candidates for consensus inclusion, based on failure to reach thresholds in one of the two studies; these are genera that bear watching as additional data are collected in future studies. Ours were the first results that placed *Malassezia* spp. (highly adapted and important commensals/pathogens of human skin) in the healthy human mouth, and built a case for its inclusion in the core oral mycobiome. Members of this genus have long been recognized as associated with numerous skin disorders and as eliciting immune responses in both commensal and pathogenic modes [34]. The role(s) that *Malassezia* species may play in oral health and disease, or in the dynamics of oral microbial communities, remains to be determined. This research, combined with others, works to build a healthy state baseline for the future study of fungal community changes in oral infections associated with immune system suppression in transplantation, chemotherapy and viral diseases.

## Materials and Methods

### Ethics Statement

Conduct of this research project with respect to human volunteers was performed according to a protocol (number X13-030) approved by the Institutional Review Board (IRB) of the University of Connecticut. The Institutional Review Board has determined that this study meets the criteria for Waiver of Informed Consent stated in 45 CFR 46.116(d).

### Saliva Collection and DNA Extraction

Volunteers were instructed to refrain from eating and drinking non-water beverages for at least one hour before donating saliva samples. While medical records and health statuses were not formally measured, all subjects were in their twenties and reported to be systemically healthy, non-smokers, and with no known oral conditions. Subjects expectorated about 3 mL of saliva into 50 mL Falcon tubes. Saliva was resuspended gently with a pipette and duplicate 1.5 mL aliquots were centrifuged at 3,300×g for 10 minutes. Supernatants were carefully removed to leave 200–300 uL and a pellet in each tube; in the case of large stringy pellets, as much supernatant as possible was removed without interfering with visible pellet material. Pellets from duplicate tubes were combined, re-pelleted, and supernatants removed to leave 200–300 uL that was extracted immediately or stored at −80°C. To extract genomic DNA (gDNA), we developed our own protocol that added ceramic beads to a standard procedure. Pellets were added to a bead beating matrix containing 0.4 g of Lysing Matrix B (MP Biomedicals, Santa Ana, California) supplemented with 1 gram of very high density 0.5 mm yttria stabilized zirconia (95% ZrO_2_ +5% Y_2_O_3_) grinding media (YSZ) to facilitate fungal cell wall and capsule breakage (Glen Mills Inc, Clifton, NJ). Next, the FastDNA SPIN KIT (MP Biomedicals, Santa Ana, California) was used according to the manufacturer's protocol with the following three modifications: 1) decreased Cell Lysis Solution for Yeast from 1000 uL to 800 uL to allow for sufficient air in the tube for thorough homogenization; 2) the single homogenization step was replaced by three homogenizations at decreased speed (5) and time (30 secs) in the FastPrep-24 Instrument (MP Biomedicals, Santa Ana, California) separated by 5 minutes on ice to keep samples cool [11]; 3) the addition of a second wash with Salt Ethanol Wash Solution to facilitate removal of lysing solutions (we found lysing solutions to persist and form precipitates in eluted gDNA if only a single wash was performed as recommended). Extracted gDNA was stored at 4°C. A negative control (reagent blank) was extracted in parallel with samples. Extraction, amplification and pyrosequencing were performed with standardly accepted sterile methods in the Center for Applied Genetics and Technology, a laboratory space designed and built specifically with segregated spaces to avoid contamination in DNA typing experiments. There were no prior experimental procedures in this space that used fungal cultures.

### Amplification and pyrosequencing of fungal ITS-1

Fusion primers containing 454 Lib-A adapter A or B, a unique 10 nucleotide multiplex identifier (MID), and forward fungal specific ITS1F primer (CTTGGTCATTTAGAGGAAGTAA [40] or reverse ITS2 primer (GCTGCGTTCTTCATCGATGC) [41] were used to amplify fungal ITS-1 sequences in triplicate (Adapters and MIDs published by Roche/454 Life Sciences). Reactions contained 125–250 ng of gDNA (measured using NanoDrop 2000, Thermo Scientific, Wilmington, DE). The average sample volume to reach 250 ng was calculated and used to determine the amount of extracted reagent blank to amplify for the negative controls. Amplification reactions were as follows: 1X OneTaq Standard Reaction Buffer, each fusion primer at 0.2 µM, 0.2 mM each dNTP, 0.025 U/µL of OneTaq Hot Start DNA Polymerase (New England BioLabs, Ipswich, MA), adjusted to a final volume of 25 µL with molecular biology grade water. The optimized thermal cycler protocol included: initial denaturation at 94°C for 30 sec; 35 cycles of denaturation at 94°C for 30 sec, annealing at 50°C for 60 sec, and extension at 68°C for 60 sec; and a final extension at 68°C for 5 min. All gDNA samples were amplified using a PTC 220 Bio-Rad Dyad Thermal Cycler (Bio-Rad Laboratories Inc, Hercules, CA). Triplicate reactions were combined and amplicon products (5 uL) initially evaluated by agarose gel electrophoresis. Remaining products were purified using the Agencourt AMPure XP System (Beckman Coulter, Inc, Indianapolis, IN) at ratios of 1.8 µL AMPure XP beads:1 µL PCR product and were eluted in 30 µL Elution buffer (Qiagen, Valencia, CA). The Pippin Prep (Sage Science, Beverly, MA) was used with 2.0% agarose gel cassettes for a size selection of 160–1000 bp to remove small primer artifacts while retaining potentially large ITS1 sequence length variants, thereby increasing productive sequencing space. Pooled samples were further cleaned with AMPure XP to remove remaining primer artifacts by one purification step at 1.6 µL beads per uL size selected sample. Quantification of amplicons and verification of primer artifact removal was accomplished with the Agilent 2100 BioAnalyzer (Agilent Technologies, Santa Clara, CA) or Experion Automated Electrophoresis System (Bio-Rad Laboratories Inc, Hercules, CA). Samples with differing MIDs were pooled in equimolar amounts and each pool also contained one parallel-processed reagent blank. Emulsion PCR was performed with the Lib-A GS FLX Titanium emPCR kit according to the manufacturer's instructions (454 Life Sciences, A Roche Company, Branford, CT). Pools were each sequenced unidirectionally on ¼ PicoTiterPlate with 454 GS FLX Titanium XLR70 reagents on the GS FLX + platform. Amplicon processing via 454 software was used to eliminate poor quality sequence reads. Raw sequencing data has been deposited in the NCBI Short Read Archive under accession number SRA107339.

### Final Data Set Curation and Taxonomic Assignments

Although the CLoVR-ITS pipeline [15] was not available at the time this work was completed, we incorporated several similar early sequence filters in our workflow. The major goal of our bioinformatics efforts was not to create a publically available automated pipeline, but rather to use an experimental and iterative process to better delineate parameters that resulted in a more concise and meaningful set of taxonomic assignments. Sequences remaining after 454 quality filtering were run through a custom bioperl script that automated a blast analysis to remove residual primer artifacts (program available upon request). The sequences were submitted to DeconSeq [42] to eliminate all sequences matching any non-18S reference databases in DeconSeq (defined by a query coverage of 90% and alignment identity of 94%). The split_libraries.py command was used in QIIME version 1.6 [43] to assign clean sequences to samples by MID and to remove sequences <100 bp after forward primer trimming. Filtering parameters were specified to allow for a maximum of 1 ambiguous base call “N”, a maximum of 10 homopolymeric repeats, a maximum of 2 barcode or forward primer mismatches, a maximum of 6 reverse primer mismatches followed by reverse primer trimming, and retention of any sequence where the reverse primer could not be found. We have implemented an additional length filter using Galaxy [44]–[46] to remove sequences of length <100 since QIIME implements length filters before removal of the reverse primer. Datasets were submitted to the Fungal Metagenomics Project (FMP) after each manipulation to assess the effects of curation on taxonomic representation. The FMP assignments were generated using a top BLAST hit without 18S masking, and the March 9, 2013 curated fungal database (updated weekly) that excluded “uncultured” reference sequences. Unassigned sequences or those assigned to taxa with FMP E-values>10^−42^ were considered poor and were separated from the curated dataset. Reliable sequence assignments (E-values≤10^−42^) were evaluated for NCBI genus using a custom perl script that accessed the matching reference sequence by GI number. Finally, genera were collapsed by hand curation using biblioinformatic guidelines suggested by Hawksworth [26]. In addition to the recommended citations in Google, Google Scholar, and Bibliography of Systemic Mycology (BSM), NIH PubMed citations were added in deference to the biomedical orientation of this research. Google searches were qualified with “fungus” when the genus names mapped to objects other than fungi (as an example, valsa refers to a waltz as well as a fungal genus). A holistic approach was used for conjoining genera. Synonyms were identified using Uniprot, BSM, and original literature. Because sexual and asexual pairs have largely been identified by binary names, we first compared species alternatives by citation numbers, weighing PubMed searches more heavily based on the health-related aspects of this research. When alternative species names had similar citation numbers, citation searches for genera were considered. Genera were not entirely conjoined unless all of the species identified in our study had synonyms in the alternative genus. To retain access to information inherent in the dual nomenclature system [27], we continued to list other genera that were collapsed into the first listed priority genus (as an example: Aspergillus/Emericella/Eurotium). When alternative genera had citation numbers that were too close for comfortably naming one as a priority designation, the original name identified by the NCBI BLAST searches was retained (as examples, Sporidiobolus and Sporobolomyces).

## Supporting Information

Table S1 **Taxonomic assignment by sequence abundance and frequency for the combined sequence dataset.**
(XLSX)

Table S2 **Incorrect Assignments of Plant-derived Amplicons to Fungal Genera.**
(XLSX)

Table S3 **Bit Scores and E-values for taxonomic assignments to subject 50.**
(XLSX)

Table S4 **Common usage survey on pairs of competing genera names.**
(DOCX)

Figure S1 **Higher order assignments for sequences unclassifiable to the level of genus.**
(DOCX)
