# Supplementary material for: Redefining the Human Oral Mycobiome with Improved Practices in Amplicon-based Taxonomy: Discovery of Malassezia as a Prominent Commensal
Source: PLoS One. 2014 Mar 10;9(3):e90899. doi: 10.1371/journal.pone.0090899 (PMC3948697; doi:10.1371/journal.pone.0090899)
Supplement: Table S4 — Common usage survey on pairs of competing genera names. (DOCX) [file pone.0090899.s004.docx]

| Table S4. Common Usage Survey on Pairs of Competing Genera Names^[[1]](#footnote-1)^ | | | | | | | | | |
| --- | --- | --- | --- | --- | --- | --- | --- | --- | --- |
| Name^[[2]](#footnote-2)^ | Google | Google Scholar | BSM | PubMed | Name**^2^** | Google | Google Scholar | BSM | PubMed |
| Trichosporon  pullulans | 7,300 | 977 |  | 32 | Guehomyces  pullulans | 7,940 | 113 |  | 7 |
| Trichosporon | 223,000 | 18,300 | 356 | 1,473 | Guehomyces | 11,400 | 130 | 5 | 8 |
|  |  |  |  |  |  |  |  | | |
| Lewia infectoria* (T) | 13,200 | 125 |  | 1 | **Alternaria infectoria (A)** | 6,140 | 471 |  | 35 |
| Chalastospora gossypii | 691 | 7 |  | 1 | **Alternaria malorum** | 3,020 | 27 |  | 3 |
| Chalastospora | 3,660 | 21 | 5 | 3 |  |  |  |  |  |
| Lewia* | 13,400 | 299 | 44 | 7 | **Alternaria** | 1,390,000 | 135,000 | 788 | 3,098 |
|  |  |  |  |  |  |  |  |  |  |
| Pichia jadinii (T) | 8,100 | 450 |  | 16 | Cyberlindnera jadinii | 84,400 | 11 |  | 0 |
| Pichia | 963,000 | 85,000 | 503 |  | Cyberlindnera | 106,000 | 30 | 1 |  |
|  |  |  |  |  | Candida utilis(A) | 120,000 | 14,500 |  | 813 |
| Debaryomyces hansenii (T) | 154,000 | 7,200 |  | 440 | Candida famata (A) | 34,500 | 2,730 |  | 130 |
| Aspergillus nidulans (A) | 501,000 | 45,200 |  | 4,079 | Emericella nidulans (T) | 90,400 | 3,160 |  | 282 |
| Aspergillus | 5,280,000 | 629,000 | 1,047 | 34,549 | Emericella | 144,000 | 5,330 | 149 | 371 |
|  |  |  |  |  |  |  |  |  |  |
| Cytospora chrysosperma | 5,580 | 795 |  | 3 | **Valsa sordida** | 5,360 | 422 |  | 5 |
| Cytospora translucens | 839 | 14 |  | 0 | **Valsa translucens** | 541 | 11 |  | 0 |
| Cytospora* (A) | 105,000 | 3,140 | 95 | 33 | **Valsa* (T)** | 424,000 | 2,230 | 131 | 61 |
|  |  |  |  |  |  |  |  |  |  |
| Filobasidium floriforme (T) | 3,310 | 187 |  | 2 | **Cryptocccus albidus (A)** | 24,600 | 3,710 |  | 177 |
| Filobasidium (T) | 8,150 | 1,120 | 93 | 31 | **Cryptococcus (A)** | 1,220,000 | 92,400 | 868 | 9,192 |
| Cystofilobasidium macerans (T) | 1,060 | 17 |  | 1 | **Cryptococcus macerans (A)** | 5,270 | 168 |  | 3 |
| Cystofilobasidium (T) | 6,830 | 571 | 75 | 45 |  |  |  |  |  |
|  |  |  |  |  |  |  |  |  |  |
| Hypocrea koningii | 11,600 | 86 |  | 0 | **Trichoderma koningii** | 37,100 | 5,590 |  | 98 |
| Hypocrea (T) | 149,000 | 5,550 | 273 | 290 | **Trichoderma (A)** | 952,000 | 160,000 | 515 | 4,083 |
|  |  |  |  |  |  |  |  |  |  |
| Polyporus mikawai | 1,170 | 13 |  | 0 | Neofavolus mikawai | 28 | 1 |  | 0 |
| Polyporus | 489,000 | 20,300 | 502 | 288 | Neofavolus | 389 | 1 | 0 | 0 |
|  |  |  |  |  |  |  |  |  |  |
|  |  |  |  |  |  |  |  |  |  |
| Peyronellaea glomerata (A) | 2,230 | 64 |  | 1 | **Phoma glomerata(A)** | 8,370 | 810 |  | 18 |
| Peyronellaea | 5,300 | 318 | 7 | 9 | **Phoma** | 792,000 | 84,500 | 665 | 425 |
| Pyrenochaetopsis pratorum | 30 | 1 |  | 0 | **Phoma pratorum (A)** | 645 | 3 |  | 0 |
| Pyrenochaetopsis | 1,980 | 9 |  | 1 |  |  |  |  |  |
|  |  |  |  |  |  |  |  |  |  |
| Talaromyces radicus (T) | 71 | 1 |  | 0 | **Penicillium radicum (A)** | 5,290 | 251 |  | 0 |
| Talaromyces | 96,500 | 7,770 | 183 | 199 | **Penicillium** | 1,400,000 | 235,000 | 989 | 10,053 |
|  |  |  |  |  |  |  |  |  |  |
| Uwebraunia commune | 180 | 5 |  | 1 | **Mycosphaerella communis** | 1,300 | 9 |  | 1 |
| Uwebraunia dekkeri | 87 | 3 |  | 1 | **Mycosphaerella lateralis (T)** | 1,960 | 56 |  | 0 |
|  |  |  |  |  |  |  |  |  |  |
| Uwebraunia | 16,200 | 56 | 16 | 1 | **Mycosphaerella** | 395,000 | 24,300 | 700 | 418 |
|  |  |  |  |  |  |  |  |  |  |
| Villosiclava virens | 10,700 | 25 |  | 2 | **Ustilaginoidea virens** | 380,000 | 1,120 |  | 18 |
| Villosiclava (T) | 13,400 | 29 | 3 | 2 | **Ustilaginoidea (A)** | 389,000 | 1,310 | 28 | 19 |
|  |  |  |  |  |  |  |  |  |  |
| Cochliobolus lunatus (T) | 36,700 | 1,140 |  | 54 | **Curvularia lunata (A)** | 56,600 | 8,210 |  | 219 |
| Cochliobolus verruculosus (T) | 2,680 | 23 |  | 0 | **Curvularia verruculosa (A)** | 1,990 | 197 |  | 4 |
| Cochliobolus (T) | 194,000 | 17,300 | 194 | 358 | **Curvularia (A)** | 185,000 | 20,100 | 265 | 549 |
|  |  |  |  |  |  |  |  |  |  |
| Coprinellus radians | 2,910 | 71 |  | 5 | **Coprinus radians** | 5,370 | 193 |  | 2 |
| Coprinellus flocculosus | 9,850 | 18 |  | 0 | **Coprinus flocculosus** | 4,580 | 27 |  |  |
| Coprinellus micaceus | 17,500 | 108 |  | 0 | **Coprinus** **micaceus** | 51,200 | 831 |  | 4 |
| Coprinellus | 59,700 | 522 | 36 | 24 | **Coprinus** | 642,000 | 20,600 | 766 | 642 |
| Coprinopsis radiata | 6,250 | 28 |  | 0 | **Coprinus radiatus** | 7,830 | 323 |  | 18 |
| Coprinopsis | 150,000 | 1,820 | 74 |  | **Obsolete?** | Synonym |  |  |  |
|  |  |  |  |  |  |  |  |  |  |
| Engyodontium album (A) | 6,320 | 278 |  | 7 | **Tritirachium album (A)** | 18,800 | 1,910 |  | 36 |
| Engyodontium | 8,450 | 416 | 36 | 14 | **Tritirachium** | 30,000 | 2,720 | 21 | 49 |
|  |  |  |  |  |  |  |  |  |  |
| Erythrobasidium hasegawianum | 3,700 | 97 |  | 8 | Rhodotorula hasegawae(A) | 1,940 | 62 |  | 3 |
| Erythrobasidium | 10,500 | 229 | 55 | 19 | Rhodotorula | 304,000 | 27,200 | 369 | 1683 |
|  |  |  |  |  |  |  |  |  |  |
| Funneliformis caledonium | 6,020 | 6 |  | 0 | **Glomus caledonium** | 19,400 | 1,040 |  | 23 |
| Funneliformis | 16,600 | 126 | 1 | 7 | **Glomus*** | 230,000 | 26,300 | 583 | 994 |
|  |  |  |  |  |  |  |  |  |  |
| Gliomastix murorum (A) | 11,300 | 419 |  | 3 | Acremonium murorum (A) | 6,740 | 194 |  | 1 |
| Gliomastix | 9,610 | 1,130 | 29 | 15 | Acremonium | 296,000 | 26,000 | 408 | 1,455 |
|  |  |  |  |  | **Synonyms** |  |  |  |  |
| Lenzites betulinus(a) | 20,701 | 1,111 |  | 15 | Trametes betulina | 1,640 | 26 |  | 0 |
| Lenzites | 64,000 | 4,050 | 147 | 27 | Trametes | 459,000 | 21,500 | 348 | 902 |
|  |  |  |  |  |  |  |  |  |  |
| Ramularia grevillana (A) | 1,200 | 17 |  | 0 | **Mycosphaerella fragariae (T)** | 11,400 | 587 |  | 3 |
| Ramularia (A) | 78,400 | 4,480 | 254 | 22 | **Synonyms** |  |  |  |  |
| Ramularia eucalypti | 271 | 5 |  | 0 |  |  |  |  |  |
|  |  |  |  |  |  |  |  |  |  |
| Discostroma fuscellum (T) | 476 | 13 |  | 0 | **Seimatosporium lichenicola** | 3,700 | 69 |  | 0 |
| Discostroma (T) | 9,130 | 145 | 37 | 1 | **Seimatosporium (A)** | 7,310 | 437 | 53 | 12 |
|  |  |  |  |  |  |  |  |  |  |
| Sporidiobolus pararoseus (T) | 3,310 | 478 |  | 11 | Sporobolomyces shibatanus (A) | 1,600 | 437 |  | 2 |
| Sporidiobolus | 17,000 | 478 | 116 | 58 | Sporobolomyces | 79,200 | 6,650 | 222 | 209 |
|  |  |  |  |  |  |  |  |  |  |
| Dioszegia hungarica | 3370 | 88 |  | 4 | **Cryptococcus hungaricus** | 3010 | 97 |  | 5 |
| Dioszegia | 6160 | 256 | 36 | 18 |  |  |  |  |  |
|  |  |  |  |  |  |  |  |  |  |
| Gibellulopsis nigrescens (A) | 9410 | 36 |  | 0 | **Verticillium nigrescens (T)** | 6280 | 297 |  | 3 |
| Gibellulopsis | 11,700 | 59 | 2 | 0 | **Verticillium** | 712,000 | 63,400 | 496 | 787 |
|  |  |  |  |  |  |  |  |  |  |

1. Searches conducted on 4/3 and 4/4 2013; Bold names are ones selected for this study. When taxa could be assigned to multiple genera, the default selection was concordance at lower taxonomic levels. [↑](#footnote-ref-1)
2. (A) indicates anamorph; (T) indicates teleomorph [↑](#footnote-ref-2)
