# Supplementary material for: Redefining the Human Oral Mycobiome with Improved Practices in Amplicon-based Taxonomy: Discovery of Malassezia as a Prominent Commensal
Source: PLoS One. 2014 Mar 10;9(3):e90899. doi: 10.1371/journal.pone.0090899 (PMC3948697; doi:10.1371/journal.pone.0090899)
Supplement: Figure S1 — Higher order assignments for sequences unclassifiable to the level of genus. (DOCX) [file pone.0090899.s005.docx]

Kingdom

Class

Phylum

Order

Family

Fungi

(7524)

Glomeromycota

(1395)

Unclassified

(3214)

Ascomycota

(2594)

Basidiomycota

(321)

Leotiomycetes

(27)

Dothideomycetes

(520)

Unclassified

(1965)

Sordariomycetes

(82)

Agaricomycetes

(182)

Exobasiodiomycetes

(6)

Unclassified

(133)

Glomeromycetes

(1395)

Unclassified

(124)

Capnodiales

(7)

Pleosporales

(389)

Xylariales

(59)

Unclassified

(13)

Hypocreales

(8)

Sordariales

(2)

Helotiales

(27)

Diversisporales

(1395)

Polyporales

(93)

Unclassified

(60)

Corticiales

(18)

Russulales

(11)

Unclassified

(6)

Unclassified

(389)

Unclassified

(7)

Unclassified

(59)

Unclassified

(8)

Unclassified

(2)

Unclassified

(27)

Unclassified

(1395)

Unclassified

(93)

Corticiaceae

(18)

Stereaceae

(11)
